# Ethical dilemmas and illicit acts in nursing: reflections on the legal (dis)order

**DOI:** 10.1590/0034-7167-2022-0558

**Published:** 2023-10-06

**Authors:** Alex Coelho da Silva Duarte, Sandra Conceição Ribeiro Chicharo, Thiago Augusto Soares Monteiro da Silva, Alexandre Barbosa de Oliveira

**Affiliations:** I Universidade Federal do Rio de Janeiro. Rio de Janeiro, Rio de Janeiro, Brazil; II Universidade Castelo Branco. Rio de Janeiro, Rio de Janeiro, Brazil

**Keywords:** Nursing, Ethics, Legislation, Nursing, Criminal Law, Jurisprudence, Enfermería, Ética, Legislación de Enfermería, Derecho Penal, Jurisprudencia, Enfermagem, Ética, Legislação de Enfermagem, Direito Penal, Jurisprudência

## Abstract

**Objectives:**

to reflect on aspects of the legal system that involve situations of ethical dilemmas and illegal acts applied in legal proceedings related to nursing professionals.

**Methods:**

theoretical-reflective essay anchored in conceptions issued by a Brazilian nursing class body, based on technical opinions, in articulation with examples extracted from judges of the Superior Court of Justice.

**Results:**

the legal sources demonstrated the need to support nursing practices through a due and clear understanding of the notions addressed. Indeed, ethical dilemmas linked to professional practice usually refer to the psychological impact of having to act differently from what feels morally, ethically, or professionally appropriate.

**Final Considerations:**

the reflection was guided by conceptual and legal issues involving nursing practice, pointing to the need to monitor the effects of legal disorder caused by current legislation, which may have implications for the legal security of professionals.

## RESUMO

**Objetivos:**

refletir sobre aspectos do ordenamento jurídico que envolvem situações de dilemas éticos e atos ilícitos aplicadas em processos judiciais relacionados a profissionais de enfermagem.

**Métodos:**

ensaio teórico-reflexivo ancorado em concepções emitidas por órgão de classe da enfermagem brasileira, embasado pelos pareceres técnicos, em articulação com exemplos extraídos de julgados do Superior Tribunal de Justiça.

**Resultados:**

as fontes jurídicas demonstraram a necessidade de se respaldarem as práticas de enfermagem por meio da compreensão devida e clara das noções abordadas. Com efeito, os dilemas éticos ligados à prática profissional geralmente se referem ao impacto psicológico de ter que agir diferente do que se sente moral, ética ou profissionalmente apropriado.

**Considerações Finais:**

a reflexão foi orientada por questões de cunho conceitual e legal que envolvem a prática de enfermagem, apontando para a necessidade de vigilância dos efeitos da desordem jurídica provocada pela legislação vigente, os quais podem trazer implicações para a segurança jurídica dos profissionais.

## RESUMEN

**Objetivos:**

reflexionar sobre aspectos del ordenamiento jurídico que implican situaciones de dilemas éticos y actos ilícitos aplicadas en procesos judiciales relacionados a profesionales de enfermería.

**Métodos:**

ensayo teórico-reflexivo ancorado en concepciones emitidas por órgano de enfermería brasileña, basado por pareceres técnicos, en articulación con ejemplos extraídos de juzgados del Superior Tribunal de Justicia.

**Resultados:**

las fuentes jurídicas demostraron la necesidad de respaldar prácticas de enfermería mediante la comprensión debida y clara de las nociones abordadas. Efectivamente, los dilemas éticos relacionados a práctica profesional generalmente referidos al impacto psicológico de tener que actuar diferente del que se siente moral, ética o profesionalmente apropiado.

**Consideraciones Finales:**

la reflexión fue orientada por cuestiones de acuño conceptual y legal que implican la práctica de enfermería, apuntando para la necesidad de vigilancia de los efectos de la desorden jurídica provocada por legislación vigente, los cuales poden traer implicaciones para la seguridad jurídica de los profesionales.

## INTRODUCTION

The ethical and legal precepts incorporated in the core of nursing practices (discipline committed to the health and quality of life of people, families, and communities) involve respect for life, dignity, and human rights in all dimensions. Such precepts function as normative provisions and rules of codes of the profession in question, adding or including certain principles, meanings, and concepts of legal jurisdiction^(1)^.

In Brazil, in addition to being a discipline of professional training and governed under the terms of higher education, nursing is a legitimate social practice and is regulated by specific legislation. Therefore, it is subject to regulatory norms and legal impositions somewhat formalized, according to ethical or deontological terms and precepts, which are also of logical and legal relevance to specific professional actions^(1)^.

Because it is still a new profession still passing through the process of being recognized, the nurse and philosopher Vilma de Carvalho, emerita professor at the Federal University of Rio de Janeiro, says that the texts of judicial proceedings have been presenting conceptual dissonance and problems involving the legal system. Given this, the reflection developed here confers with plausible expositions, especially about the arguments and justifications that help in the understanding of professional activities, to add more clarity in the legal approach to illegal acts (negligence, malpractice, and imprudence) and especially the ethical dilemmas related to the actions of nursing professionals.

In the context of contemporary ethical problems, it is a priority to discuss the use of the term “dilemma,” especially concerning the particularities and intersections of the fields of nursing and law. This theme refers to the award-winning literary work “Sofia’s choice,” by William Styron, in 1979, in which a mother, a prisoner in the Auschwitz concentration camp during World War II, receives a cruel order to choose which of his two sons should be executed and which should remain alive^(2)^. The work frames the dilemma as a problem that has, as a rule, two solutions: one about what is allowed to do and the other about what the personal feeling, at the moment faced, indicates to do^(2)^. The two solutions end up not being complete as a whole: behold, between what can be done and what one wants to do, some doubts end up arising.

On the polysemic word “ethics,” the Brazilian philosopher Mário Sérgio Cortella considers it synthetically as:

the set of values and principles we use to answer three big questions in life: (1) Do I want to? (2) should I? and (3) can I? For not all that I want, I can; not all that I can, I must; and not everything I should, I want. You have peace of mind when what you want is both what you can and what you should^(3)^.

When articulating these two terms, “ethical dilemmas,” we understand that they refer to the psychological impact of having to act other than what feels morally, ethically, or professionally appropriate^(4)^. Ethical dilemmas are related to everyday issues present in the work of nursing professionals, who face sensitive situations in their practice in the context of interpersonal relationships between professionals and patients-families-communities.

Indeed, the ethical processes related to nursing practices established within the scope of oversight bodies of the profession have revealed a high frequency of infractions committed predominantly by professionals at the beginning of their careers, most commonly nursing technicians and those in activities that are not within their competence or that involve illicit acts of negligence, malpractice, and imprudence^(5)^. This perspective demarcates the need for (re)knowledge of current legislation, the proper use of certain notions and concepts that bring potential implications in the legal field, but also a critical and well-considered (re)vision of the legal system regarding the competencies of nursing professionals in the country.

Based on the approach of the notion of “ethical dilemmas,” it is also necessary to clarify why dissatisfaction with the profession and labor problems themselves do not correspond to what is understood by an ethical dilemma. Thus, delays in rendering, accumulation of service, lack of personnel in the sector, and personal issues with other professionals are not legally understood as ethical dilemmas since these do not put vulnerability in the decision-making capacity between what can and what should be done. It is also necessary to pacify the idea that unintentional error (willful deception) due to malpractice, recklessness, and/or negligence should not be characterized as ethical dilemmas, but infractions subject to deontological, civil, and criminal punishment.

This imbroglio is constantly observed in legal texts, including technical ones, which report cases involving nursing professionals, which is a sensitive aspect of this study. Thus, it is based on the premise that nursing professionals need to work according to the laws governing the profession, ensuring the well-being of the team, the necessary items for their protection, as well as the patients assistance, avoiding damage to human and collective health. It demands (re)knowledge of their legislation to advance the ethical/citizen consciousness and political-social responsibility that are embedded in their practices. Therefore, the understanding and reflection on the legally correct use of the notions mentioned here seek to help in the approach of certain ethical-legal aspects related to the specific activities of the profession in the complex universe of health while demonstrating the need to consider the legal system that considers such activities.

## OBJECTIVES

To reflect on aspects of the legal system that involve situations of ethical dilemmas and illegal acts applied in legal proceedings related to nursing professionals.

## METHODS

This is a theoretical-reflective essay anchored in conceptions issued by the Cofen/Coren system responsible for the supervision of the nursing profession in Brazil, based on the technical opinions of this system in conjunction with examples extracted from judges of the Superior Court of Justice (STJ).

For its development, cases with decisions in the last three years (from 2020 to 2022) were accessed, using the keywords “nursing,” “imprudence,” “malpractice,” and “negligence,” in the electronic address: https://www.stj.jus.br. In total, it identified 1,059 cases, of which, after full reading, selected three that fit the objective set for this study.

The discussion was guided by the use of the Conglobant Typicity Theory of Eugenio Raúl Zaffaroni^(6)^, which highlights the need to observe the legal system as a whole to typify conduct as anti-legal, i.e., the conglobant typicity demonstrates what is prohibited by a specific legal device, seeking to avoid, in this way, that a norm prohibits what another norm orders or promotes.

The text is structured in a single thematic axis, namely: ethical dilemmas and illicit acts in the field of nursing and legal issues.

## RESULTS

### Ethical dilemmas and illicit acts in the field of Nursing and legal issues

To approach the notions of recklessness, malpractice, and negligence as illegal acts, those put in the technical opinion 03/2020 of the Regional Nursing Council of the Federal District - Coren-DF are listed, with examples extracted from decisions of the STJ:

Recklessness is rash conduct. While, in a negligence situation, the error is in the omission (not doing), in recklessness, the error is precisely in the activities conducted, but without the due caution and wisdom that the situation requires. The risk involved is known, but security measures are either not taken or are conducted without the necessary rigor. That is, the nursing team exercises its care practices without the due care that the situation requires^(1)^.

To exemplify the legal application concerning recklessness, this study mention a case of an accident that occurred in the classroom. It is the decision of Minister Ricardo Villas Bôas Cueva, in which a nursing undergraduate student who volunteers to play a victim during a practical first aid class and ends up accidentally hit by another student, suffering severe injuries, as shown in the following highlights:

It is an incontrovertible fact in the records that the applicant was a student of the Nursing Course at the requested University and suffered the accident in the classroom during the practical class of said nursing course on 04/16/2017.

During the practical class, the student had to lie down on the ground to demonstrate first aid, and another student, who was also a volunteer in the demonstration, ended up falling on the appellant-author’s hand, which caused her too much pain, requiring medical assistance, as reported by the parties and confirmed by the witnesses (p. 664-696).

The applicant also demonstrates that she was diagnosed with post-traumatic Reflex Sympathetic Dystrophy since 04/16/17, which would have been caused by the aforementioned accident that occurred in the classroom (p. 39). The author was unable to work and exercise her activities for a long period in 2007 and 2008 due to problems with her right hand (p. 35-51). So much so that she even received a sick pay benefit, given his incapacitation from work (p. 52-59).

The witnesses reported that there were about twenty (20) students in the room at the time of the accident and that the teacher divided the class into groups, but with poorly organization. They narrated that they were not warned to maintain a certain safety distance or about the necessary precautions and possible risks of the simulation. They considered that it was difficult for the teacher to dominate a class with so many students, especially in a practical class. They maintain that the author became a joke in the class because of the accident and the resulting physical limitation (p. 673-682 and 682-691)^(7)^.

The highlighted excerpt, according to the witnesses’ report, reveals that the teacher taught the practical class without specific precautions to the characteristics of the educational intervention being taken, i.e., the students were not guided and warned about the potential risks involved in the development of that practical activity.

Believing that the teacher in question had previous professional practice and competence to assume such responsibility, by assumption, she knew the risk involved but, as evidenced, did not take the appropriate safety measures with the rigor that the situation required, configuring her recklessness, which led to the mentioned accident.

Imprudence is distinguished from malpractice, as the latter, there is a lack of qualification to perform a specific action or procedure, as can be seen below:

Malpractice refers to a lack of technical skill. As in situations of recklessness, when there is malpractice, the condemnable act is in the action and not in the omission. Malpractice is checked when an activity is conducted by a professional without proper qualification and training, theoretical or practical. He/she is taking a risk on himself/herself and others. Malpractice generates civil and criminal liability for the professional who performed the actions^(1)^.

The decision of Minister Humberto Martins of the STJ brings a clear example of malpractice, as can be seen from the prominence of the decision:

[...] there was a medical indication for the preservation of the right upper limb, considering the presence of hemangioma at the site. Nevertheless, the medical advice was not observed by the nursing team, a fact that led to an ulcerated lesion in the right upper limb of the minor author due to extravasation of calcium chloride serum, which culminated in a scar of 10 cm long by 2 cm wide. In the complementary report, the expert confirmed the medical determination to preserve the right upper limb, avoiding puncture of this segment. (mov. 189.1).

Nevertheless, analyzing the medical record of the minor, it is found that there was a puncture in venous access in the right upper limb of the minor (mov. 24.13).

Therefore, the hospital’s nursing team failed to comply with medical advice, which resulted in physical damage to the minor (ulcerated necrotic lesion).

In addition, as the expert pointed out, in addition to disobedience to medical advice, there was malpractice of the hospital nursing team, configured by the extravasation of glucose serum and destruction of the arteriovenous fistula (p. 933/934)^(7)^.

In addition to the non-observance of the express guidance, as can be seen from the excerpts, there was the infusion of venous fluids in a non-proper route, the arteriovenous fistula, which characterized the lack of qualification and training of members of that nursing team, that is, its malpractice.

Concerning the notion of negligence, the following excerpt reports aspects to be highlighted:

Negligence is the lack of due attention, the result of the omission of the individual (professional), as well as the passivity in a situation that causes a particular result, whereas this professional should conduct some action. Some definitions also consider the lack of care or inattention in the execution of a particular task as negligence, as well as indifference^(6)^.

To exemplify the use of this notion, the following excerpt from the decision of Minister Humberto Martins, also from the STJ, stands out:

...However, the nursing staff would be responsible for letting patient “S” (mother of “M” and “R”, and the author’s spouse, “J”), fall from her height during a bath that was prescribed to her. This negligence is the great stimulus of the present action, the subject of a police report, made while the patient was still alive, where she, represented by her daughter, narrates that she was put to bathe alone because the wheelchair was not suitable to be wet, so they took her out of the chair and put her on her feet in the shower box, balancing on one leg since the other had lost in an accident, which is why she fell injuring her spleen and fracturing her femur. Nothing that compromises the conduct of the defendant doctor, Dr. “F”. Only, the nurses’, for whom the hospital is responsible...^(7)^.

In this case, the judge was guided by an expert report (opinion issued by a medical professional appointed to the case), which emphatically stressed the failure of the nursing staff: “... because, in the case of a patient with only one lower limb, she could not have been left alone to take a bath or another activity inside the room. The risk of falling was predictable”^(7)^.

Despite the inconsistency of the example extracted from the jurisprudence, the negligence of the members of the nursing team was characterized and, unless better judgment, clear from the ethical-legal point of view, although the expert report is issued by a professional without the due technical competence to evaluate nursing service, as provided for by the current law on professional practice in Brazil.

In the legal aspect, the notion of ethical-legal dilemmas can be highlighted as a complement to what has been presented so far. For better exemplification, it is considered a recent situation of socio-environmental disaster that occurred in the municipality of Petrópolis, located in the mountainous region of the state of Rio de Janeiro, at the beginning of 2022, in which a voluminous rainfall soaked the ground and caused several landslides, resulting in the collapse of structures and buildings and the burial of several people. It is an extreme situation in which lives can be lost in a second, and professionals readily employed, or volunteers are responsible for carrying out specific advanced life support procedures on victims, many believing they are legally based on their code of professional ethics, which expressly points out the care of critically ill patients with life-threatening conditions among its attributions.

However, this is sometimes against Law 12,842/2013, which defines such a procedure as exclusive to another professional category, and Federal Council of Medicine (CFM) resolution 1,718/2004. Moreover, this legal instrument prevents them from learning certain care practices, providing that “the teaching of private medical acts, in any form of transmission of knowledge, to non-medical professionals, including those relevant to advanced health care support, is prohibited, except the emergency care at a distance, until optimal resources are achieved”^(8-9)^.

In contradiction, this resolution provides that, in case of emergencies, this “teaching” is allowed in the telemedicine modality until the optimal resources are achieved. In conclusion, the nurse cannot have prior knowledge of the advanced procedures but, in case of emergencies/disasters, this professional can learn how to perform them with authorization/prescription virtually through telemedicine^(10)^.

This legal disorder puts the nursing professional in a legal-ethical dilemma in which, on the one hand, will be law 12.842 of 2013, known as the “law of the medical act,” and on the other, life. Therefore, questions for reflection are raised here, such as: “is it plausible to wait for the arrival of moments of chaos, in which lives depend on immediate intervention, to then be taught specific assistance procedures that can, in fact, save lives? When a disaster happens, all local care capacity usually has been exhausted, and complementary reinforcements need to be activated, is it possible to apply care techniques that demonstrably make the difference between life and death”? Consequently, when considering the psychic aspects directly and indirectly involved in such extreme situations, it is believed that this is not the most appropriate time to be taught emergency life-saving and maintenance techniques^(8)^.

At this point, it is appropriate to present the ideas of the jurist, professor, and judge of the Inter-American Court of Human Rights, Eugenio Raúl Zaffaroni, who, together with the jurist and prosecutor of the state of São Paulo, José Henrique Pierangelli, developed the “Conglobant Typicity Theory.”^(9)^ This theory comprises that it cannot be considered as “typical” a conduct that is previously and expressly permitted by the legal system. For the authors, the legal system is sole one, global, covering all areas of law (civil, criminal, commercial, procedural etc.). Therefore, no one can practice a typical fact (crime) if an action allowed by the legal order global is performed^(9)^.

When reflecting on the example presented above (of an advanced life support procedure) and based on it consider the aspects of the legal system, we understand that the maintenance of life is being opposed to the normative act that prohibits the teaching of procedures considered exclusive to doctors, however, which can be performed by non-medical professionals in cases of particular situations of emergencies and disasters.

This prohibitive norm is in opposition to all legal systems, and this situation ends up raising doubts, real ethical-legal dilemmas for nursing professionals. It reinforces the need to establish greater legal certainty for actions in cases of emergencies and disasters, for example.

As assumed by the Conglobant Typicity Theory, an act cannot be classified as a crime if it is allowed by another norm constant in the legal system of a given country. In addition, the judgment of typicity imposes, in addition to the legal typicity, the conglobant typicity, which consists in ascertaining the prohibitive scope of the norm, which cannot be taken in isolation, but conglobated in the legal order^(9)^.

Notably, ethical dilemmas involve other everyday situations in which the nurse practitioner needs to make a specific decision, usually choosing to act other than what feels morally, ethically, or professionally appropriate. Many of these ethical dilemmas will be directly related to professional practice and will arise from the legal disorder caused by the legislation itself, sometimes divergent, on the same subject. In these cases, one must be borne in mind that life is the legal asset that seeks to preserve and, among the fundamental rights provided for in the Brazilian Federal Constitution, this is undoubtedly the most important because, without life, one cannot enjoy the other rights provided for.

In addition, regarding the Conglobant Typicity Theory, which provides for the analysis of all legal system to characterize the typicity of conduct, the legal disorder can be combated and, consequently, achieve greater legal certainty in the actions of nursing professionals. It is because, currently, there are no legal instruments in the country to give legal support to particular interventions, such as public health emergencies and disasters.

Thus, it is necessary to consider that criminal dogmatics in Brazil has suffered harsh sociological criticism accompanying the idea of the delegitimization of the criminal system, understanding that if it has lost legitimacy, there is no support for criminal dogmatics^(6)^.

This is a sober reason to firmly consider how it should be the safe and fair way to better frame contemporary nursing practices in a satisfactory legal system and under a prism of intellectual honesty, which duly supports its professionals to observe the governing foundations of the profession and ethical-humanistic values. Therefore, nursing professionals need to be strongly articulated with the organs of class representation in the assumption of effective and well-sustained strategies.

## FINAL CONSIDERATIONS

To guarantee rights, it is necessary to (re)know the laws since they regulate life in society and also guide professional acts; in addition, the notions and concepts that typify, through the ethical spectrum, certain practices must be properly employed. Based on this, the analysis addressed the need to demarcate the specificities of the notions of ethical dilemmas and cases of malpractice, imprudence and negligence since these cases are punishable infractions and not situations in which a decision is questioned in the face of legality in acting in a certain way, as ethical dilemmas are.

The reflection on the use of such notions, in the light of judicial decisions of the STJ, and especially the understanding of the problems of legal order in the national scenario, have the effect of providing nursing professionals with greater decision-making capacity, ethical and legal support for the exercise of their functions without suffering ethical, civil, and criminal sanctions inherent to the position. In addition, they provide the necessary critical review of their legal actions and predictions.

Finally, there is a need to develop new studies on the researched theme, especially those situations not yet properly framed by the available professional legislation, since the profusion of public policies, technologies, practices and innovations in care and management does not always accompany the legal movement of (re)definition of competencies in the face of the new challenges imposed on the nursing profession.
